# *N*′-[(*E*)-1-Phenyl­ethyl­idene]benzo­hydrazide

**DOI:** 10.1107/S1600536808029218

**Published:** 2008-09-20

**Authors:** Hoong-Kun Fun, K. V. Sujith, P. S. Patil, B. Kalluraya, Suchada Chantrapromma

**Affiliations:** a X-ray Crystallography Unit, School of Physics, Universiti Sains Malaysia, 11800 USM, Penang, Malaysia; b Department of Studies in Chemistry, Mangalore University, Mangalagangotri, Mangalore 574 199, India; c Department of Physics, K.L.E. Society’s K.L.E. Institute of Technology, Gokul Road, Hubli 590 030, India; d Crystal Materials Research Unit, Department of Chemistry, Faculty of Science, Prince of Songkla University, Hat-Yai, Songkhla 90112, Thailand

## Abstract

The title compound, C_15_H_14_N_2_O, crystallized with two independent mol­ecules in the asymmetric unit. Both mol­ecules are non-planar and have an *E* configuration with respect to the C=N bond. The dihedral angles between the two benzene rings are 11.1 (2)° in one mol­ecule and 12.40 (19)° in the other. In the crystal structure, the mol­ecules are linked by N—H⋯O hydrogen bonds and weak C—H⋯O inter­actions into infinite one-dimensional chains along [1 0 0]. The crystal structure is further stabilized by N—H⋯O hydrogen bonds, weak C—H⋯O and very weak C—H⋯π inter­actions.

## Related literature

For bond-length data, see: Allen *et al.* (1987 ▶). For background to the applications of hydrazone and benzohydrazide, see, for example: Bratenko *et al.* (1999 ▶); Raj *et al.* (2007 ▶); Rollas *et al.* (2002 ▶); Sridhar *et al.* (2003 ▶); Zhang *et al.* (2007 ▶).
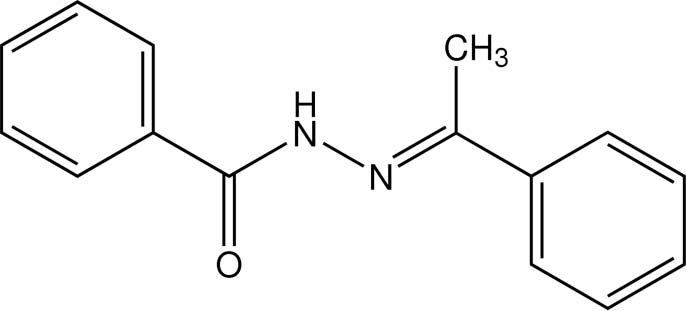


## Experimental

### 

#### Crystal data

C_15_H_14_N_2_O
*M* *_r_* = 238.28
Orthorhombic, 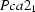

*a* = 8.2237 (6) Å
*b* = 5.5938 (4) Å
*c* = 52.839 (4) Å
*V* = 2430.7 (3) Å^3^
*Z* = 8
Mo *K*α radiation
μ = 0.08 mm^−1^
*T* = 100.0 (1) K
0.50 × 0.22 × 0.05 mm

#### Data collection

Bruker APEXII CCD area-detector diffractometer
Absorption correction: multi-scan (*SADABS*; Bruker, 2005 ▶) *T*_min_ = 0.959, *T*_max_ = 0.996
23699 measured reflections
3572 independent reflections
2925 reflections with *I* > 2σ(*I*)
*R*_int_ = 0.069

#### Refinement

*R*[*F* ^2^ > 2σ(*F* ^2^)] = 0.064
*wR*(*F* ^2^) = 0.159
*S* = 1.08
3572 reflections
327 parameters
1 restraint
H-atom parameters constrained
Δρ_max_ = 0.29 e Å^−3^
Δρ_min_ = −0.30 e Å^−3^

### 

Data collection: *APEX2* (Bruker, 2005 ▶); cell refinement: *SAINT* (Bruker, 2005 ▶); data reduction: *SAINT*; program(s) used to solve structure: *SHELXTL* (Sheldrick, 2008 ▶); program(s) used to refine structure: *SHELXTL*; molecular graphics: *SHELXTL* software used to prepare material for publication: *SHELXTL* and *PLATON* (Spek, 2003 ▶).

## Supplementary Material

Crystal structure: contains datablocks global, I. DOI: 10.1107/S1600536808029218/si2108sup1.cif

Structure factors: contains datablocks I. DOI: 10.1107/S1600536808029218/si2108Isup2.hkl

Additional supplementary materials:  crystallographic information; 3D view; checkCIF report

## Figures and Tables

**Table 1:** Hydrogen-bond geometry (Å, °)

*D*—H⋯*A*	*D*—H	H⋯*A*	*D*⋯*A*	*D*—H⋯*A*
N2*A*—H2*NA*⋯O2*A*^i^	0.91	1.95	2.857 (4)	170
N2*B*—H2*NB*⋯O2*B*^ii^	0.73	2.17	2.866 (4)	161
C14*A*—H14*A*⋯O2*A*^i^	0.93	2.38	3.108 (5)	135
C14*B*—H14*B*⋯O2*B*^ii^	0.93	2.38	3.113 (5)	135
C15*A*—H15*A*⋯O2*A*^i^	0.96	2.58	3.053 (5)	110
C15*B*—H15*D*⋯O2*B*^ii^	0.96	2.44	3.038 (5)	120
C1*A*—H1*AA*⋯*Cg*1^ii^	0.93	2.96	3.729 (4)	141
C4*A*—H4*AA*⋯*Cg*1^iii^	0.93	2.95	3.724 (5)	141
C1*B*—H1*BA*⋯*Cg*2^i^	0.93	2.88	3.726 (4)	141
C4*B*—H4*BA*⋯*Cg*2^iv^	0.93	2.94	3.714 (4)	141

Symmetry codes: (i) 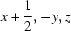; (ii) 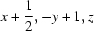; (iii) 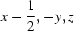; (iv) 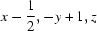. *Cg*1 and *Cg*2 are the centroids of the C1*A*–C6*A* and C1*B*–C6*B* phenyl rings, respectively.

**Fig. 1.**
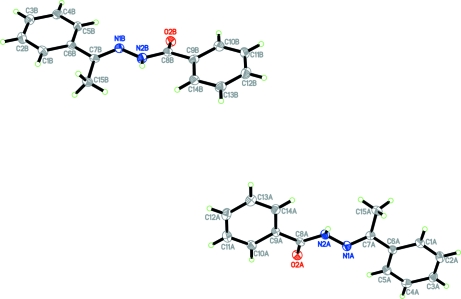
The asymmetric unit of (I), showing 50% probability displacement ellipsoids and the atomic numbering.

**Fig. 2.**
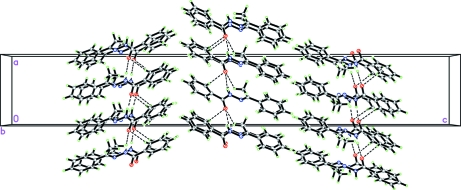
The crystal packing of the major components of (I), viewed along the b axis showing that the molecules are linked in infinite one-dimensional chains along the a axis. Hydrogen bonds are drawn as dashed lines.

## supplementary crystallographic information

### Comment

Hydrazones are versatile intermediates and important building blocks. Hydrazones of aliphatic and aromatic methyl ketones yield pyrazole-4-carboxaldehyde upon diformylation on treatment with Vilsmeier reagent (Bratenko *et al.*, 1999). Aryl hydrazones are important building blocks for the synthesis of a variety of heterocyclic compounds such as pyrazolines and pyrazoles (Sridhar *et al.*, 2003). Aryl hydrazones have been most conveniently synthesized by the reaction of aryl hydrazines with carbonyl compounds. Hydrazones have been demonstrated to possess antimicrobial, anticonvulsant, analgesic, antiinflammatory, antiplatelet, antitubercular, anticancer and antitumoral activities (Rollas *et al.*, 2002). Hydrazones possessing an azometine -NHN=CH- proton constitute an important class of compounds for new drug development. Therefore, many researchers have synthesized these compounds as well as their metal complexes as target structures and evaluated their biological activities (Raj *et al.*, 2007; Zhang *et al.*, 2007). These observations guided us to synthesize the title compound and its crystal structure was reported here.

In the asymmetric unit of the title compound (Fig. 1), there are two independent molecules *A* and *B*. Bond lengths in molecules *A* and *B* are slightly different but all are in normal ranges (Allen *et al.*, 1987). Both molecules are not planar and exist in the *E* configuration which respect to the C═N bond. The dihedral angles between the two benzene rings are 11.1 (2)° in *A* and 12.40 (19)° in *B*. In molecule *A*, the interplanar angle between the mean plane through N2A/O2A/C8A/C9A and N1A/N2A/C6A/C7A/C15A planes = 20.8 (2)°. In molecule *B* atoms N1B, N2B, C7B and C15B lie on the same plane and this plane makes the dihedral angle with the mean plane through N2B/O2B/C8B/C9B = 20.4 (2)°.

Fig. 2 shows that the molecules are linked into chains along [1 0 0] through N—H···O hydrogen bonds and weak C—H···O and very weak C—H···π interactions (Table 1); *Cg*_1_, *Cg*_2_, *Cg*_3_ and *Cg*_4_ are the centroids of the C1A–C6A, C1B–C6B, C9A–C14A and C9B–C14B rings, respectively.

### Experimental

The title compound was obtained by refluxing phenyl hydrazide (0.01 mol) and acetophenone (0.01 mol) in ethanol (30 ml) by adding 3 drops of concentrated Sulfuric acid for 3 hr. Excess ethanol was removed from the reaction mixture under reduced pressure. The solid product obtained was filtered, washed with ethanol and dried. Colorless single crystals of the title compound suitable for *x*-ray structure determination were grown by slow evaporation of an ethanol solution at room temperature.

### Refinement

All H atoms were constrained in a riding motion approximation with N—H = 0.73 and 0.91 Å, C_aryl_—H=0.93 and C_methyl_—H=0.96 Å. The *U*_iso_ values were constrained to be 1.5*U*_eq_ of the carrier atom for methyl H atoms and 1.2*U*_eq_ for the remaining H atoms. A rotating group model was used for the methyl groups. The highest residual electron density peak is located at 0.76 Å from C10A and the deepest hole is located at 0.72 Å from H1AA. As there is no large anomalous dispersion for the determination of the absolute structure, a total of 2250 Friedel pairs were merged before final refinement.

### Crystal data

**Table:**

C_15_H_14_N_2_O	*F*(000) = 1008
*M**_r_* = 238.28	*D*_x_ = 1.302 Mg m^−^^3^
Orthorhombic, *P**c**a*2_1_	Mo *K*α radiation, λ = 0.71073 Å
Hall symbol: P 2c -2ac	Cell parameters from 3572 reflections
*a* = 8.2237 (6) Å	θ = 0.8–30.0°
*b* = 5.5938 (4) Å	µ = 0.08 mm^−^^1^
*c* = 52.839 (4) Å	*T* = 100 K
*V* = 2430.7 (3) Å^3^	Plate, colorless
*Z* = 8	0.50 × 0.22 × 0.05 mm

### Data collection

**Table:**

Bruker SMART APEXII CCD area-detector diffractometer	3572 independent reflections
Radiation source: fine-focus sealed tube	2925 reflections with *I* > 2σ(*I*)
graphite	*R*_int_ = 0.069
Detector resolution: 8.33 pixels mm^-1^	θ_max_ = 30.0°, θ_min_ = 0.8°
ω scans	*h* = −11→11
Absorption correction: multi-scan (SADABS; Bruker, 2005)	*k* = −7→7
*T*_min_ = 0.959, *T*_max_ = 0.996	*l* = −74→73
23699 measured reflections	

### Refinement

**Table:**

Refinement on *F*^2^	Primary atom site location: structure-invariant direct methods
Least-squares matrix: full	Secondary atom site location: difference Fourier map
*R*[*F*^2^ > 2σ(*F*^2^)] = 0.064	Hydrogen site location: inferred from neighbouring sites
*wR*(*F*^2^) = 0.159	H-atom parameters constrained
*S* = 1.08	*w* = 1/[σ^2^(*F*_o_^2^) + (0.0598*P*)^2^ + 2.0097*P*] where *P* = (*F*_o_^2^ + 2*F*_c_^2^)/3
3572 reflections	(Δ/σ)_max_ < 0.001
327 parameters	Δρ_max_ = 0.29 e Å^−^^3^
1 restraint	Δρ_min_ = −0.30 e Å^−^^3^

### Special details

**Table:**

Experimental. The low-temperature data was collected with the Oxford Cyrosystem Cobra low-temperature attachment.
Geometry. All e.s.d.'s (except the e.s.d. in the dihedral angle between two l.s. planes) are estimated using the full covariance matrix. The cell e.s.d.'s are taken into account individually in the estimation of e.s.d.'s in distances, angles and torsion angles; correlations between e.s.d.'s in cell parameters are only used when they are defined by crystal symmetry. An approximate (isotropic) treatment of cell e.s.d.'s is used for estimating e.s.d.'s involving l.s. planes.
Refinement. Refinement of *F*^2^ against ALL reflections. The weighted *R*-factor *wR* and goodness of fit *S* are based on *F*^2^, conventional *R*-factors *R* are based on *F*, with *F* set to zero for negative *F*^2^. The threshold expression of *F*^2^ > σ(*F*^2^) is used only for calculating *R*-factors(gt) *etc*. and is not relevant to the choice of reflections for refinement. *R*-factors based on *F*^2^ are statistically about twice as large as those based on *F*, and *R*- factors based on ALL data will be even larger.

### Fractional atomic coordinates and isotropic or equivalent isotropic displacement parameters (Å^2^)

**Table:**

	*x*	*y*	*z*	*U*_iso_*/*U*_eq_	
O2A	0.4444 (3)	−0.2422 (5)	0.28192 (5)	0.0225 (5)	
N1A	0.5650 (4)	0.0608 (6)	0.24842 (6)	0.0211 (7)	
N2A	0.6306 (4)	0.0417 (6)	0.27253 (6)	0.0201 (7)	
H2NA	0.7358	0.0912	0.2746	0.024*	
C1A	0.5851 (5)	0.4279 (7)	0.19173 (7)	0.0228 (8)	
H1AA	0.6561	0.5481	0.1968	0.027*	
C2A	0.5277 (5)	0.4246 (7)	0.16709 (7)	0.0248 (8)	
H2AA	0.5604	0.5425	0.1558	0.030*	
C3A	0.4219 (5)	0.2471 (7)	0.15916 (7)	0.0237 (8)	
H3AA	0.3846	0.2445	0.1426	0.028*	
C4A	0.3719 (6)	0.0726 (7)	0.17624 (8)	0.0259 (8)	
H4AA	0.2996	−0.0457	0.1711	0.031*	
C5A	0.4294 (5)	0.0744 (7)	0.20083 (7)	0.0221 (8)	
H5AA	0.3959	−0.0440	0.2120	0.026*	
C6A	0.5372 (5)	0.2515 (6)	0.20916 (7)	0.0183 (7)	
C7A	0.6028 (4)	0.2496 (6)	0.23548 (7)	0.0186 (7)	
C8A	0.5670 (5)	−0.1290 (6)	0.28767 (7)	0.0194 (7)	
C9A	0.6454 (5)	−0.1709 (6)	0.31291 (7)	0.0192 (7)	
C10A	0.6062 (5)	−0.3840 (7)	0.32523 (7)	0.0237 (8)	
H10A	0.5381	−0.4942	0.3174	0.028*	
C11A	0.6686 (6)	−0.4318 (7)	0.34912 (8)	0.0281 (9)	
H11A	0.6405	−0.5727	0.3574	0.034*	
C12A	0.7725 (5)	−0.2705 (7)	0.36069 (8)	0.0284 (9)	
H12A	0.8161	−0.3045	0.3765	0.034*	
C13A	0.8115 (5)	−0.0584 (7)	0.34857 (7)	0.0264 (8)	
H13A	0.8798	0.0509	0.3565	0.032*	
C14A	0.7494 (5)	−0.0079 (7)	0.32479 (7)	0.0223 (7)	
H14A	0.7768	0.1343	0.3167	0.027*	
C15A	0.7015 (5)	0.4561 (6)	0.24455 (8)	0.0240 (8)	
H15A	0.6978	0.4627	0.2627	0.036*	
H15B	0.6581	0.6016	0.2377	0.036*	
H15C	0.8121	0.4370	0.2391	0.036*	
O2B	0.7470 (3)	0.7474 (4)	0.48915 (5)	0.0219 (5)	
N1B	0.8664 (4)	0.4422 (5)	0.52262 (6)	0.0191 (6)	
N2B	0.9356 (4)	0.4640 (6)	0.49876 (6)	0.0217 (7)	
H2NB	1.0056	0.4027	0.4934	0.026*	
C1B	0.8836 (5)	0.0735 (7)	0.57897 (7)	0.0230 (8)	
H1BA	0.9549	−0.0459	0.5738	0.028*	
C2B	0.8256 (5)	0.0752 (7)	0.60357 (7)	0.0256 (8)	
H2BA	0.8590	−0.0419	0.6149	0.031*	
C3B	0.7171 (5)	0.2523 (7)	0.61138 (7)	0.0242 (8)	
H3BA	0.6778	0.2529	0.6279	0.029*	
C4B	0.6682 (5)	0.4275 (7)	0.59443 (7)	0.0233 (8)	
H4BA	0.5953	0.5450	0.5996	0.028*	
C5B	0.7273 (5)	0.4279 (6)	0.56996 (7)	0.0214 (8)	
H5BA	0.6950	0.5472	0.5588	0.026*	
C6B	0.8357 (5)	0.2499 (6)	0.56181 (7)	0.0178 (7)	
C7B	0.9020 (5)	0.2528 (6)	0.53547 (7)	0.0181 (7)	
C8B	0.8716 (5)	0.6352 (6)	0.48358 (7)	0.0191 (7)	
C9B	0.9556 (5)	0.6867 (7)	0.45879 (7)	0.0200 (7)	
C10B	0.9161 (5)	0.9028 (7)	0.44669 (8)	0.0239 (8)	
H10B	0.8449	1.0098	0.4543	0.029*	
C11B	0.9835 (5)	0.9560 (7)	0.42336 (7)	0.0248 (8)	
H11B	0.9575	1.0990	0.4153	0.030*	
C12B	1.0899 (5)	0.7967 (7)	0.41187 (7)	0.0258 (8)	
H12B	1.1345	0.8333	0.3962	0.031*	
C13B	1.1300 (5)	0.5833 (7)	0.42368 (7)	0.0249 (8)	
H13B	1.2033	0.4785	0.4161	0.030*	
C14B	1.0592 (5)	0.5268 (7)	0.44704 (7)	0.0228 (8)	
H14B	1.0821	0.3809	0.4547	0.027*	
C15B	1.0038 (5)	0.0472 (6)	0.52656 (7)	0.0215 (8)	
H15D	1.0135	0.0531	0.5085	0.032*	
H15E	0.9531	−0.1004	0.5314	0.032*	
H15F	1.1099	0.0571	0.5341	0.032*	

### Atomic displacement parameters (Å^2^)

**Table:**

	*U*^11^	*U*^22^	*U*^33^	*U*^12^	*U*^13^	*U*^23^
O2A	0.0164 (13)	0.0242 (12)	0.0268 (13)	−0.0029 (11)	−0.0018 (11)	0.0001 (10)
N1A	0.0199 (17)	0.0221 (15)	0.0214 (15)	−0.0012 (12)	−0.0012 (13)	0.0022 (12)
N2A	0.0191 (17)	0.0198 (15)	0.0213 (16)	−0.0015 (12)	−0.0014 (13)	0.0019 (12)
C1A	0.023 (2)	0.0184 (16)	0.027 (2)	−0.0005 (14)	0.0004 (15)	0.0014 (14)
C2A	0.023 (2)	0.0258 (18)	0.0253 (19)	0.0017 (16)	−0.0004 (16)	0.0067 (15)
C3A	0.025 (2)	0.0271 (19)	0.0189 (17)	0.0047 (16)	−0.0020 (15)	0.0016 (14)
C4A	0.029 (2)	0.0225 (18)	0.0258 (18)	−0.0018 (16)	−0.0003 (16)	−0.0034 (15)
C5A	0.0199 (19)	0.0209 (17)	0.0254 (18)	−0.0009 (15)	0.0002 (15)	0.0020 (14)
C6A	0.0164 (17)	0.0182 (15)	0.0203 (16)	0.0023 (13)	0.0006 (13)	0.0000 (13)
C7A	0.0143 (17)	0.0199 (16)	0.0216 (17)	0.0029 (13)	−0.0002 (13)	−0.0012 (13)
C8A	0.0195 (18)	0.0158 (15)	0.0229 (18)	0.0009 (14)	0.0000 (15)	0.0001 (13)
C9A	0.0168 (18)	0.0188 (16)	0.0221 (17)	0.0005 (13)	0.0011 (14)	0.0027 (13)
C10A	0.025 (2)	0.0204 (17)	0.0261 (19)	0.0000 (15)	−0.0029 (16)	0.0017 (14)
C11A	0.028 (2)	0.0273 (19)	0.029 (2)	0.0009 (16)	0.0015 (17)	0.0061 (16)
C12A	0.028 (2)	0.034 (2)	0.0226 (18)	0.0027 (17)	−0.0020 (16)	0.0043 (16)
C13A	0.021 (2)	0.033 (2)	0.0253 (19)	−0.0016 (16)	0.0004 (16)	−0.0012 (15)
C14A	0.022 (2)	0.0233 (16)	0.0218 (18)	−0.0040 (16)	0.0001 (16)	0.0010 (14)
C15A	0.024 (2)	0.0192 (17)	0.029 (2)	0.0007 (15)	−0.0032 (16)	−0.0005 (14)
O2B	0.0192 (13)	0.0213 (12)	0.0253 (13)	0.0023 (11)	0.0021 (10)	0.0007 (10)
N1B	0.0182 (16)	0.0200 (14)	0.0193 (15)	−0.0017 (12)	0.0029 (12)	−0.0006 (11)
N2B	0.0213 (17)	0.0247 (15)	0.0190 (15)	0.0021 (13)	0.0058 (13)	−0.0008 (12)
C1B	0.0215 (19)	0.0189 (16)	0.028 (2)	0.0017 (14)	0.0004 (16)	0.0025 (15)
C2B	0.025 (2)	0.0252 (18)	0.027 (2)	0.0000 (16)	−0.0021 (17)	0.0070 (15)
C3B	0.022 (2)	0.0281 (18)	0.0221 (18)	−0.0019 (15)	0.0016 (15)	0.0002 (15)
C4B	0.024 (2)	0.0255 (18)	0.0210 (17)	0.0030 (16)	0.0015 (15)	−0.0030 (14)
C5B	0.026 (2)	0.0183 (15)	0.0205 (17)	0.0028 (14)	−0.0004 (15)	0.0012 (13)
C6B	0.0158 (17)	0.0142 (14)	0.0235 (16)	−0.0021 (13)	0.0002 (14)	0.0006 (12)
C7B	0.0155 (17)	0.0153 (16)	0.0237 (18)	−0.0002 (13)	−0.0012 (13)	−0.0028 (13)
C8B	0.0188 (18)	0.0181 (16)	0.0204 (17)	−0.0015 (14)	0.0005 (14)	−0.0018 (13)
C9B	0.0177 (18)	0.0219 (16)	0.0204 (16)	−0.0029 (14)	0.0015 (14)	−0.0035 (14)
C10B	0.0217 (19)	0.0229 (17)	0.027 (2)	0.0011 (15)	−0.0024 (15)	0.0000 (15)
C11B	0.028 (2)	0.0220 (18)	0.0240 (18)	−0.0030 (15)	−0.0011 (16)	0.0041 (14)
C12B	0.025 (2)	0.0319 (19)	0.0201 (17)	−0.0053 (16)	0.0006 (15)	0.0014 (15)
C13B	0.025 (2)	0.0270 (17)	0.0222 (18)	0.0012 (16)	0.0045 (16)	−0.0037 (14)
C14B	0.025 (2)	0.0201 (16)	0.0231 (18)	0.0018 (15)	−0.0015 (15)	−0.0001 (14)
C15B	0.0212 (19)	0.0185 (16)	0.0250 (18)	0.0035 (14)	0.0011 (15)	−0.0018 (14)

### Geometric parameters (Å, °)

**Table:**

O2A—C8A	1.229 (5)	O2B—C8B	1.237 (5)
N1A—C7A	1.296 (5)	N1B—C7B	1.292 (5)
N1A—N2A	1.388 (4)	N1B—N2B	1.389 (4)
N2A—C8A	1.351 (5)	N2B—C8B	1.356 (5)
N2A—H2NA	0.9145	N2B—H2NB	0.7271
C1A—C2A	1.385 (5)	C1B—C2B	1.384 (5)
C1A—C6A	1.406 (5)	C1B—C6B	1.397 (5)
C1A—H1AA	0.9300	C1B—H1BA	0.9300
C2A—C3A	1.385 (6)	C2B—C3B	1.395 (6)
C2A—H2AA	0.9300	C2B—H2BA	0.9300
C3A—C4A	1.392 (6)	C3B—C4B	1.387 (5)
C3A—H3AA	0.9300	C3B—H3BA	0.9300
C4A—C5A	1.382 (5)	C4B—C5B	1.381 (5)
C4A—H4AA	0.9300	C4B—H4BA	0.9300
C5A—C6A	1.400 (5)	C5B—C6B	1.404 (5)
C5A—H5AA	0.9300	C5B—H5BA	0.9300
C6A—C7A	1.492 (5)	C6B—C7B	1.495 (5)
C7A—C15A	1.491 (5)	C7B—C15B	1.498 (5)
C8A—C9A	1.499 (5)	C8B—C9B	1.509 (5)
C9A—C10A	1.396 (5)	C9B—C14B	1.382 (5)
C9A—C14A	1.399 (5)	C9B—C10B	1.406 (5)
C10A—C11A	1.388 (6)	C10B—C11B	1.384 (6)
C10A—H10A	0.9300	C10B—H10B	0.9300
C11A—C12A	1.385 (6)	C11B—C12B	1.389 (6)
C11A—H11A	0.9300	C11B—H11B	0.9300
C12A—C13A	1.386 (6)	C12B—C13B	1.387 (6)
C12A—H12A	0.9300	C12B—H12B	0.9300
C13A—C14A	1.385 (5)	C13B—C14B	1.401 (5)
C13A—H13A	0.9300	C13B—H13B	0.9300
C14A—H14A	0.9300	C14B—H14B	0.9300
C15A—H15A	0.9600	C15B—H15D	0.9600
C15A—H15B	0.9600	C15B—H15E	0.9600
C15A—H15C	0.9600	C15B—H15F	0.9600
			
C7A—N1A—N2A	117.0 (3)	C7B—N1B—N2B	117.1 (3)
C8A—N2A—N1A	116.6 (3)	C8B—N2B—N1B	116.1 (3)
C8A—N2A—H2NA	120.6	C8B—N2B—H2NB	114.2
N1A—N2A—H2NA	117.0	N1B—N2B—H2NB	129.5
C2A—C1A—C6A	120.7 (4)	C2B—C1B—C6B	120.5 (4)
C2A—C1A—H1AA	119.6	C2B—C1B—H1BA	119.8
C6A—C1A—H1AA	119.6	C6B—C1B—H1BA	119.8
C3A—C2A—C1A	120.5 (4)	C1B—C2B—C3B	120.2 (4)
C3A—C2A—H2AA	119.7	C1B—C2B—H2BA	119.9
C1A—C2A—H2AA	119.7	C3B—C2B—H2BA	119.9
C2A—C3A—C4A	119.5 (4)	C4B—C3B—C2B	119.7 (4)
C2A—C3A—H3AA	120.3	C4B—C3B—H3BA	120.1
C4A—C3A—H3AA	120.3	C2B—C3B—H3BA	120.1
C5A—C4A—C3A	120.2 (4)	C5B—C4B—C3B	120.2 (4)
C5A—C4A—H4AA	119.9	C5B—C4B—H4BA	119.9
C3A—C4A—H4AA	119.9	C3B—C4B—H4BA	119.9
C4A—C5A—C6A	121.1 (4)	C4B—C5B—C6B	120.6 (3)
C4A—C5A—H5AA	119.4	C4B—C5B—H5BA	119.7
C6A—C5A—H5AA	119.4	C6B—C5B—H5BA	119.7
C5A—C6A—C1A	117.9 (3)	C1B—C6B—C5B	118.8 (3)
C5A—C6A—C7A	121.1 (3)	C1B—C6B—C7B	120.6 (3)
C1A—C6A—C7A	121.0 (3)	C5B—C6B—C7B	120.6 (3)
N1A—C7A—C15A	126.3 (3)	N1B—C7B—C6B	114.5 (3)
N1A—C7A—C6A	114.3 (3)	N1B—C7B—C15B	126.2 (3)
C15A—C7A—C6A	119.4 (3)	C6B—C7B—C15B	119.2 (3)
O2A—C8A—N2A	122.4 (3)	O2B—C8B—N2B	122.6 (3)
O2A—C8A—C9A	119.5 (3)	O2B—C8B—C9B	119.3 (3)
N2A—C8A—C9A	118.1 (3)	N2B—C8B—C9B	118.1 (3)
C10A—C9A—C14A	119.2 (3)	C14B—C9B—C10B	119.7 (4)
C10A—C9A—C8A	116.7 (3)	C14B—C9B—C8B	123.3 (3)
C14A—C9A—C8A	124.1 (3)	C10B—C9B—C8B	116.9 (3)
C11A—C10A—C9A	120.2 (4)	C11B—C10B—C9B	119.8 (4)
C11A—C10A—H10A	119.9	C11B—C10B—H10B	120.1
C9A—C10A—H10A	119.9	C9B—C10B—H10B	120.1
C12A—C11A—C10A	120.2 (4)	C10B—C11B—C12B	120.3 (4)
C12A—C11A—H11A	119.9	C10B—C11B—H11B	119.9
C10A—C11A—H11A	119.9	C12B—C11B—H11B	119.9
C11A—C12A—C13A	119.7 (4)	C13B—C12B—C11B	120.4 (4)
C11A—C12A—H12A	120.1	C13B—C12B—H12B	119.8
C13A—C12A—H12A	120.1	C11B—C12B—H12B	119.8
C14A—C13A—C12A	120.6 (4)	C12B—C13B—C14B	119.4 (4)
C14A—C13A—H13A	119.7	C12B—C13B—H13B	120.3
C12A—C13A—H13A	119.7	C14B—C13B—H13B	120.3
C13A—C14A—C9A	120.0 (4)	C9B—C14B—C13B	120.4 (4)
C13A—C14A—H14A	120.0	C9B—C14B—H14B	119.8
C9A—C14A—H14A	120.0	C13B—C14B—H14B	119.8
C7A—C15A—H15A	109.5	C7B—C15B—H15D	109.5
C7A—C15A—H15B	109.5	C7B—C15B—H15E	109.5
H15A—C15A—H15B	109.5	H15D—C15B—H15E	109.5
C7A—C15A—H15C	109.5	C7B—C15B—H15F	109.5
H15A—C15A—H15C	109.5	H15D—C15B—H15F	109.5
H15B—C15A—H15C	109.5	H15E—C15B—H15F	109.5
			
C7A—N1A—N2A—C8A	167.6 (3)	C7B—N1B—N2B—C8B	166.7 (3)
C6A—C1A—C2A—C3A	0.0 (6)	C6B—C1B—C2B—C3B	0.7 (6)
C1A—C2A—C3A—C4A	0.7 (6)	C1B—C2B—C3B—C4B	−0.3 (6)
C2A—C3A—C4A—C5A	−1.0 (6)	C2B—C3B—C4B—C5B	−0.5 (6)
C3A—C4A—C5A—C6A	0.6 (6)	C3B—C4B—C5B—C6B	0.9 (6)
C4A—C5A—C6A—C1A	0.1 (6)	C2B—C1B—C6B—C5B	−0.3 (6)
C4A—C5A—C6A—C7A	−178.3 (3)	C2B—C1B—C6B—C7B	178.1 (4)
C2A—C1A—C6A—C5A	−0.4 (6)	C4B—C5B—C6B—C1B	−0.5 (6)
C2A—C1A—C6A—C7A	178.0 (3)	C4B—C5B—C6B—C7B	−178.9 (4)
N2A—N1A—C7A—C15A	−4.2 (6)	N2B—N1B—C7B—C6B	174.9 (3)
N2A—N1A—C7A—C6A	176.2 (3)	N2B—N1B—C7B—C15B	−3.6 (6)
C5A—C6A—C7A—N1A	6.8 (5)	C1B—C6B—C7B—N1B	−170.8 (3)
C1A—C6A—C7A—N1A	−171.5 (3)	C5B—C6B—C7B—N1B	7.6 (5)
C5A—C6A—C7A—C15A	−172.8 (4)	C1B—C6B—C7B—C15B	7.8 (5)
C1A—C6A—C7A—C15A	8.8 (5)	C5B—C6B—C7B—C15B	−173.8 (3)
N1A—N2A—C8A—O2A	−9.0 (5)	N1B—N2B—C8B—O2B	−8.4 (5)
N1A—N2A—C8A—C9A	174.2 (3)	N1B—N2B—C8B—C9B	172.6 (3)
O2A—C8A—C9A—C10A	19.1 (5)	O2B—C8B—C9B—C14B	−158.2 (4)
N2A—C8A—C9A—C10A	−164.0 (3)	N2B—C8B—C9B—C14B	20.9 (6)
O2A—C8A—C9A—C14A	−158.9 (4)	O2B—C8B—C9B—C10B	17.6 (5)
N2A—C8A—C9A—C14A	18.0 (5)	N2B—C8B—C9B—C10B	−163.3 (3)
C14A—C9A—C10A—C11A	0.6 (6)	C14B—C9B—C10B—C11B	−1.2 (6)
C8A—C9A—C10A—C11A	−177.4 (4)	C8B—C9B—C10B—C11B	−177.2 (4)
C9A—C10A—C11A—C12A	−1.2 (6)	C9B—C10B—C11B—C12B	0.0 (6)
C10A—C11A—C12A—C13A	1.4 (6)	C10B—C11B—C12B—C13B	−0.2 (6)
C11A—C12A—C13A—C14A	−1.1 (6)	C11B—C12B—C13B—C14B	1.5 (6)
C12A—C13A—C14A—C9A	0.5 (6)	C10B—C9B—C14B—C13B	2.6 (6)
C10A—C9A—C14A—C13A	−0.3 (6)	C8B—C9B—C14B—C13B	178.3 (4)
C8A—C9A—C14A—C13A	177.6 (4)	C12B—C13B—C14B—C9B	−2.7 (6)

### Hydrogen-bond geometry (Å, °)

**Table:**

*D*—H···*A*	*D*—H	H···*A*	*D*···*A*	*D*—H···*A*
N2A—H2NA···O2A^i^	0.91	1.95	2.857 (4)	170
N2B—H2NB···O2B^ii^	0.73	2.17	2.866 (4)	161
C14A—H14A···O2A^i^	0.93	2.38	3.108 (5)	135
C14B—H14B···O2B^ii^	0.93	2.38	3.113 (5)	135
C15A—H15A···N2A	0.96	2.47	2.811 (5)	100
C15A—H15A···O2A^i^	0.96	2.58	3.053 (5)	110
C15B—H15D···N2B	0.96	2.44	2.812 (5)	103
C15B—H15D···O2B^ii^	0.96	2.45	3.038 (5)	120
C1A—H1AA···Cg1^ii^	0.93	2.96	3.729 (4)	141
C4A—H4AA···Cg1^iii^	0.93	2.95	3.724 (5)	141
C1B—H1BA···Cg2^i^	0.93	2.88	3.726 (4)	141
C10A—H10A···Cg3^iv^	0.93	3.31	3.993 (4)	132
C10B—H10B···Cg4^v^	0.93	3.16	3.847 (4)	132
C4B—H4BA···Cg2^vi^	0.93	2.94	3.714 (4)	141
C13A—H13A···Cg3^i^	0.93	3.05	3.674 (4)	126
C13B—H13B···Cg4^ii^	0.93	3.07	3.755 (4)	132

Symmetry codes: (i) *x*+1/2, −*y*, *z*; (ii) *x*+1/2, −*y*+1, *z*;  (iii) *x*−1/2, −*y*, *z*; (iv) *x*−1/2, −*y*−1, *z*; (v) *x*−1/2, −*y*+2, *z*; (vi) *x*−1/2, −*y*+1, *z*.
